# Machine learning‐driven GWAS uncovers novel candidate genes for resistance to frosty pod rot and witches' broom disease in cacao

**DOI:** 10.1002/tpg2.70069

**Published:** 2025-07-10

**Authors:** Ezekiel Ahn, Sunchung Park, Insuck Baek, Dongho Lee, Jishnu Bhatt, Seunghyun Lim, Jae Hee Jang, Dapeng Zhang, Moon S. Kim, Lyndel W. Meinhardt

**Affiliations:** ^1^ USDA‐ARS Sustainable Perennial Crops Laboratory Beltsville Maryland USA; ^2^ USDA‐ARS Agriculture, Environmental Microbial and Food Safety Laboratory Beltsville Maryland USA; ^3^ USDA‐ARS Environmental Microbial and Food Safety Laboratory Beltsville Maryland USA

## Abstract

Cacao (*Theobroma cacao*), the source of chocolate, is threatened by devastating diseases like frosty pod rot (FPR) and witches' broom disease (WBD), impacting global production and farmer livelihoods. Here, we employ a machine learning‐driven genome‐wide association study to dissect the genetic architecture of disease resistance and productivity in cacao. Upon analyzing phenotypic data for healthy pod rate along with FPR and WBD resistance across 102 diverse accessions, coupled with single nucleotide polymorphism (SNP) markers mapped to the Criollo and Matina reference genomes, we identified numerous novel candidate genes. These genes are implicated in various biological processes, including cell wall modification, stress response signaling, and defense‐related mechanisms. Notably, associations varied between the reference genomes, highlighting the genomic complexity of these traits. Our analyses, using Bootstrap Forest and Boosted Tree models, uncovered loci not previously reported, demonstrating the power of machine learning in uncovering complex genetic interactions. This study offers important insights into the polygenic nature of disease resistance in cacao and presents a genomic roadmap for developing disease‐resistant varieties.

## Core Ideas

- Machine learning genome‐wide association studies (GWAS) identified novel genes for frosty pod rot (FPR) and witches' broom disease (WBD) resistance in cacao.
- Candidate genes implicate cell wall, stress response, and defense in polygenic disease resistance.
- Varied associations in Criollo and Matina genomes portray complexity of resistance traits.
- This study provides genomic roadmap to breed cacao for resistance to diseases like frosty pod rot (FPR) and WBD.
- Combined GWAS results demonstrate diverse processes in cacao productivity and disease resistance.

**Abbreviations**
AUDPC: area under the disease progress curve
BF: Bootstrap Forest
BT: Boosted Tree
FPR: frosty pod rot
GBS: genotyping‐by‐sequencing
GO: Gene Ontology
GWAS: genome‐wide association study
IBS: identity by state
MAS: marker‐assisted selection
ML: machine learning
PC1: first principal component
PCA: principal component analysis
QTL: quantitative trait locus
SNP: single nucleotide polymorphism
t‐SNE: t‐distributed stochastic neighbor embedding
WBD: witches' broom disease

## 1 INTRODUCTION

As the source of cocoa, the globally significant perennial tree crop *Theobroma cacao* L. underpins the multi‐billion dollar chocolate industry and supports the livelihoods of millions of smallholder farmers in developing nations, significantly contributing to their national economies (Díaz‐Montenegro, 2019; Hall, 2022). However, many small‐scale producers face economic challenges that threaten the long‐term sustainability of their operations (Manrique et al., 2024). Achieving a competitive advantage in the global market is crucial for the long‐term viability of the cacao agro‐industry, particularly in regions like Southeast Sulawesi, Indonesia, where factors such as market orientation and supply chain flexibility play a significant role (Hatani et al., 2016). Beyond its economic importance, sustainable cacao cultivation contributes to biodiversity conservation and carbon sequestration (Obeng & Aguilar, 2015). The specialty cacao industry, in particular, has been recognized for its potential to enhance farmer welfare and environmental resource conservation through premium pricing and direct trade models (Cadby & Araki, 2021). However, the future of cacao production faces mounting challenges, including the impacts of climate change (Ceccarelli et al., 2021), volatile market prices (Villacis et al., 2022), and the persistent threat of devastating diseases (Evans, 2007). Addressing these challenges is paramount, not only for ensuring the long‐term viability of the chocolate industry but also for safeguarding the livelihoods of farmers and promoting sustainable development in cacao‐growing regions.

Among biotic constraints, diseases exert significant pressure on cacao productivity worldwide. Two of the most economically impactful diseases are frosty pod rot (FPR), caused by the hemibiotrophic fungus *Moniliophthora roreri*, and witches' broom disease (WBD), caused by *Moniliophthora perniciosa* (Phillips‐Mora et al., 2007; Purdy & Schmidt, 1996). FPR is characterized by its destructive pod rot and has been reported to cause yield losses of up to 80% in some regions, making it a major threat to cacao production in Latin America (Phillips‐Mora et al., 2007). WBD, a systemic disease affecting various plant tissues, causes severe yield decline and, in extreme cases, tree mortality. WBD, first described in the late 1700s, has a long history of impacting cacao production in South America and presents ongoing management challenges (Purdy & Schmidt, 1996). Since its initial description in the late 18th century, WBD has caused dramatic shifts in cacao production, including Brazil's fall from a leading exporter to a net importer following a severe outbreak in the late 1980s (Meinhardt et al., 2008).

Traditional disease management approaches rely heavily on cultural practices, sanitation, and occasional fungicide use; unfortunately, these methods often prove insufficient or too costly to implement, particularly for resource‐constrained smallholder farmers (Purdy & Schmidt, 1996). Therefore, the development and deployment of disease‐resistant cacao varieties are increasingly recognized as a critical and sustainable solution for mitigating the impact of FPR and WBD. Harnessing genetic resistance offers a powerful and environmentally sound strategy for managing cacao diseases (Poland et al., 2012). While sources of resistance to both FPR and WBD have been identified within the vast and largely untapped genetic diversity of cacao germplasm, the introgression of these traits into elite, high‐yielding varieties through conventional breeding remains a slow and arduous process that often takes decades (Bartley, 2005; Gutiérrez et al., 2021; McElroy et al., 2018; Osorio‐Guarín et al., 2020; Romero Navarro et al., 2017). Additionally, understanding the genetic diversity and biogeography of pathogens like *M. roreri* is crucial for developing effective and durable resistance strategies (Phillips‐Mora et al., 2007).

The emergence of genomics, however, has revolutionized plant breeding, providing unprecedented opportunities to accelerate the development of improved varieties (Lahive et al., 2019). The release of the first cacao reference genome (Criollo) in 2011 (Argout et al., 2017), followed by a second (Matina) in 2013 (Motamayor et al., 2013), has established fundamental genomic resources for cacao research, paving the way for marker‐assisted selection (MAS) and other genomic‐assisted breeding approaches. While MAS holds immense potential for enhancing the efficiency and precision of cacao breeding programs, challenges to its effective implementation remain, particularly in managing QTL stability and integrating MAS into traditional breeding practices (Hospital, 2009).

Genome‐wide association studies (GWAS) have become a cornerstone of modern plant genetics, providing a powerful means to dissect the complex genetic architecture of polygenic traits (Brachi et al., 2011). By leveraging high‐density single nucleotide polymorphism (SNP) markers distributed across the entire genome, GWAS can identify genomic regions and, ultimately, candidate genes that contribute to phenotypic variation (Brachi et al., 2011). In cacao, GWAS has been applied to identify loci associated with yield components, bean quality, aroma, and resistance to specific diseases (Colonges et al., 2022; Wickramasuriya & Dunwell, 2018). The availability of the cacao genome sequences has facilitated these efforts, enabling the development of high‐density SNP arrays and the identification of candidate genes. However, previous studies have often been constrained by limited scope, focusing primarily on single diseases or narrow germplasm collections.

A more holistic approach is crucial for a comprehensive understanding of the genetic groundwork of these complex traits. This should encompass a broader spectrum of genetic diversity and a simultaneous examination of multiple traits related to productivity and stress resistance, including both abiotic (e.g., drought and heat) and biotic stresses (e.g., pathogen‐triggered stress) (Lahive et al., 2019). Recent research has highlighted the polygenic nature of traits such as WBD resistance, identifying multiple QTLs with minor effects and revealing the intricate host‐pathogen interactions involved (Chia Wong et al., 2022). For instance, Romero Navarro et al. (2017) applied GWAS and genomic prediction to a population of improved cacao clones, locating markers associated with resistance against FPR and black pod rot as well as yield‐related traits. Similarly, Osorio‐Guarín et al. (2020) conducted a GWAS on a diverse collection of 102 cacao accessions to ascertain candidate genes for productivity and disease resistance using traditional statistical methods.

Building on this foundational work, our study employed advanced machine learning (ML) techniques, including Bootstrap Forest (BF) (Random Forest) and Boosted Tree (BT) models (Breiman, 2001; Chen, 2014), to re‐analyze the phenotypic and genotypic data generated by Osorio‐Guarín et al. (2020). These methods are particularly suited to handle high‐dimensional data and capturing complex interactions between genes and environmental factors. We further conducted a comparative analysis using both the Criollo and Matina reference genomes to assess the influence of reference genome choice on SNP discovery and association mapping. We hypothesized that the genetic architecture of productivity and disease resistance in cacao involves numerous loci with small‐to‐moderate effects and that the choice of reference genome can significantly impact SNP‐trait associations. By integrating GWAS with advanced ML techniques, we aimed to provide a deeper understanding of the genetic basis of healthy pod rate and resistance to FPR and WBD. The results of this comprehensive study provide valuable new insights for cacao breeding programs and serve as a valuable resource for future functional validation studies.

**Core Ideas**

- Machine learning genome‐wide association studies (GWAS) identified novel genes for frosty pod rot (FPR) and witches' broom disease (WBD) resistance in cacao.
- Candidate genes implicate cell wall, stress response, and defense in polygenic disease resistance.
- Varied associations in Criollo and Matina genomes portray complexity of resistance traits.
- This study provides genomic roadmap to breed cacao for resistance to diseases like frosty pod rot (FPR) and WBD.
- Combined GWAS results demonstrate diverse processes in cacao productivity and disease resistance.

## 2 MATERIALS AND METHODS

### 2.1 Genetic distance and phenotypic correlation analysis

To investigate the relationship between genetic relatedness and phenotypic variation among the 229 cacao accessions, we performed a correlation analysis using genetic distances and phenotypic data. Genetic distances were estimated using the identity by state (IBS) method implemented in the TASSEL 5 software (Bradbury et al., 2007), which calculated the proportion of alleles shared between each pair of individuals across all marker loci, providing a measure of genetic similarity. Two sets of genome‐wide SNP markers were used for this analysis: 9003 SNPs mapped to the Criollo reference genome (Argout et al., 2017) and 8131 SNPs mapped to the Matina reference genome (Motamayor et al., 2013). These SNP datasets were obtained from the genotyping‐by‐sequencing (GBS) data previously generated and described by Osorio‐Guarín et al. (2020). As reported for this dataset mapped to the Criollo genome, the average distance between markers was 39.8 kb, ranging from 31.1 to 43.3 kb depending on the chromosome (Osorio‐Guarín et al., 2020). The IBS calculations resulted in two separate distance matrices, denoted as “IBS‐Matina” and “IBS‐Criollo,” corresponding to the reference genome used for SNP mapping.

Phenotypic data for four traits of 102 accessions related to cacao productivity and disease resistance were also obtained from Osorio‐Guarín et al. (2020). These traits included: (1) healthy pod rate, calculated as the number of healthy pods divided by the total number of pods for each accession; (2) pods with FPR, quantified using the area under the disease progress curve (AUDPC) to reflect the severity of FPR caused by *M. roreri*; (3) flower with cushion WBD, representing the AUDPC of flower cushions infected with WBD caused by *M. perniciosa*; and (4) branches with WBD, representing the AUDPC of branches exhibiting WBD symptoms. The AUDPC was calculated as described by Shaner and Finney (1977).

Pearson's correlation coefficients were computed between each genetic distance matrix (IBS‐Matina and IBS‐Criollo) and each of the four phenotypic traits using the JMP Pro 17 software (SAS Institute Inc.; Klimberg, 2023). This analysis assessed the degree to which genetic similarity, as measured by IBS, was associated with phenotypic similarity for each trait.

### 2.2 Hierarchical clustering analysis

To explore the structure within the genotypic and phenotypic data, we performed hierarchical clustering analyses using JMP Pro 17. Two distinct clustering approaches were employed. First, we conducted genotype‐based clustering separately for each reference genome (Criollo and Matina) using the SNP datasets described previously. For each reference genome, a distance matrix was calculated based on the SNP data using the Ward method (Murtagh & Legendre, 2014), an agglomerative hierarchical clustering algorithm implemented in JMP Pro 17. Second, we performed clustering based on a combination of genotype and phenotype data. The phenotypic data for the four traits (healthy pod rate, FPR, flower cushions with WBD, and branches with WBD) were combined with the SNP data for each reference genome (Criollo and Matina). A distance matrix was calculated for each combined dataset (Criollo SNPs + phenotypes, Matina SNPs + phenotypes) using the Ward method in JMP Pro 17. The Ward method (Murtagh & Legendre, 2014) was then applied to cluster the accessions based on their combined genotypic and phenotypic profiles.

### 2.3 t‐Distributed stochastic neighbor embedding analysis

To further explore and visualize the population structure within the genotypic data, we performed t‐distributed stochastic neighbor embedding (t‐SNE) analyses using JMP Pro 17. t‐SNE is a nonlinear dimensionality reduction technique that aims to preserve local neighborhood relationships in high‐dimensional data when reducing it to a lower dimensional representation, typically two or three dimensions (Hamid & Sugumaran, 2020). Separate t‐SNE analyses were conducted for each reference genome (Criollo and Matina) using the corresponding SNP data. Analyses were performed using JMP Pro 17′s default settings, which include a perplexity value of 30, a learning rate (epsilon) of 10, and a maximum of 1,000 iterations.

### 2.4 Genome‐wide association study using ML

To identify genomic regions associated with variation in four phenotypic traits (healthy pod rate, pods exhibiting FPR, flower cushions with WBD, and branches affected by WBD), GWAS were conducted using two ML algorithms: BF and BT, implemented in JMP Pro 17. These tree‐based ensemble methods were chosen for their potential ability to capture nonlinear relationships and complex interactions (epistasis) between SNPs, aspects of genetic architecture that may not be fully accounted for by standard additive linear models often used in GWAS. These analyses were carried out separately for each trait based on SNP data mapped to both the Criollo and Matina reference genomes. Population structure, such as principal components or an estimated Q‐matrix, was not explicitly included as a covariate in these models. For the BF method, we used the following settings: number of trees in the forest = 100, number of terms sampled per split = 1 (this is the square root of the number of predictors, rounded down, and is the default setting), bootstrap sample rate = 1, minimum splits per tree = 10, maximum splits per tree = 2000, and minimum size split = 5. For the BT method, we employed the following settings: learning rate = 0.1, minimum size split = 5, maximum layers = 6, number of layers to estimate = 20, and sampling rate = 1. The importance of each SNP in predicting the trait of interest was assessed using the “Portion” statistic, representing the relative contribution of each SNP to the overall model precision, with higher values indicating greater importance. The results of these analyses are visualized in Manhattan plots, which display the logarithm of the exponentiated Portion scores (Log‐Exp(Importance score)) for each SNP along the respective chromosomes/scaffolds. For identifying candidate regions associated with each trait and model combination, SNPs were ranked based on their importance scores (“Portion”). The top five SNPs with the highest importance scores were selected for further investigation and candidate gene identification (Tables 1, 2, 3 and Table S1).

**TABLE 1:** Candidate genes associated with cacao productivity and disease resistance identified by top single nucleotide polymorphisms (SNPs) from Bootstrap Forest genome‐wide association studies (GWAS) using the Criollo reference genome.

Trait	Chr	SNP position	Nearest genes and function	GO annotation	Importance score (Portion)
Bootstrap Forest					
Healthy pod rate	5	7754541, 7754552	*Tc05v2_g008540* Secoisolariciresinol dehydrogenase (7730282–7731554)	–	0.0317
	3	3271174	*Tc03v2_g003210* Uncharacterized protein isoform 2 (3301477–3307744)	Cellular component, membrane	0.0252
	8	13135179	*Tc08v2_g013610* Beta‐fructofuranosidase, insoluble isoenzyme 1 (12892301–12893596)	–	0.019
	5	10063324	*Tc05v2_g009360* Uncharacterized protein (10072093–10084247)	–	0.0147
	5	20378993	*Tc05v2_g010930* Gag protease polyprotein‐like protein (20381149–20381871)	–	0.0129
Pods with FPR	3	25139428	*Tc03v2_g009640* Probable galactinol–sucrose galactosyltransferase 2 (25135345–25139665)	–	0.0643
	9	23881358	*Tc09v2_g017680* DEAD‐box ATP‐dependent RNA helicase 37 (23889018–23895419)	Hydrolase activity, mRNA binding, ATP binding	0.0278
	1	18355124, 18285303, 18354976	*Tc01v2_g019210* Uncharacterized protein (18311296–18312102)	–	0.0201
	1	36944112	*Tc01v2_g034430* Uncharacterized protein (36937551–36945789)	–	0.0158
	6	823888	*Tc06v2_g000820* Putative lectin (814417–818887)	–	0.0156
Flower with cushion WBD	2	770436	***Tc02v2_g001090*** Probable xyloglucan galactosyltransferase GT17 (768191–770560)	–	0.1528
	2	1064432	***Tc02v2_g001650*** Ion channel DMI1 (1060675–1066653)	–	0.142
	1	21174545, 21174462	*Tc01v2_g019770* Ferrochelatase‐2, chloroplastic (21204419–21219868)	–	0.061
	1	31472241	*Tc01v2_g025400* Putative exopolygalacturonase clone GBGE184 (31467814–31469699)	–	0.0498
	2	4523324	***Tc02v2_g007330*** MLO‐like protein 4 (4521274–4527984)	–	0.0272
Branches with WBD	6	10714823	*Tc06v2_g005450* Putative NAC domain‐containing protein 35 (10785856–10788539)	Transcription regulation, DNA binding	0.0361
	9	1133400	*Tc09v2_g001870* Topless‐related protein 1 (1136104–1143084)	–	0.0237
	10	2544954	*Tc10v2_g004160* Cysteine/Histidine‐rich C1 domain family protein, putative (2556402–2564757)	–	0.0204
	7	3689811, 3689626	*Tc07v2_g005490* G‐type lectin S‐receptor‐like serine/threonine‐protein kinase At4g27290 (3679662–3684095)	–	0.0167
	10	19646083	*Tc10v2_g012370* Putative cytosolic sulfotransferase 15 (19634059–19635764)	–	0.0128
Boosted Tree					
Healthy pod rate	5	7754541	*Tc05v2_g008540* Secoisolariciresinol dehydrogenase (7730282–7731554)	–	0.1168
	8	13135179	*Tc08v2_g013610* Beta‐fructofuranosidase, insoluble isoenzyme 1 (12892301–12893596)	–	0.0943
	5	9516723	*Tc05v2_g009250* Uncharacterized protein (9552088–9552591)	–	0.0725
	7	10954840	*Tc07v2_g012560* Uncharacterized protein (10937178–10937777)	–	0.0709
	3	3271174	*Tc03v2_g003210* Uncharacterized protein isoform 2 (3301477–3307744)	Cellular Component, membrane	0.059
Pods with FPR	9	23881358	*Tc09v2_g017680* DEAD‐box ATP‐dependent RNA helicase 37 (23889018–23895419)	Hydrolase activity, mRNA binding, ATP binding	0.1818
	6	6853962	*Tc06v2_g004520* Long chain base biosynthesis protein 1 (6864200–6873051)	–	0.1615
	1	24715162	*Tc01v2_g020390* Uncharacterized protein (24734066–24734969)	Cellular component, membrane	0.0821
	3	25139428	*Tc03v2_g009640* Probable galactinol–sucrose galactosyltransferase 2 (25135345–25139665)	–	0.0744
	1	36944112	*Tc01v2_g034430* Uncharacterized protein (36937551–36945789)	–	0.038
Flower with cushion WBD	2	770436	***Tc02v2_g001090*** Probable xyloglucan galactosyltransferase GT17 (768191–770560)	–	0.2104
	2	1064432	***Tc02v2_g001650*** Ion channel DMI1 (1060675–1066653)	–	0.2054
	1	21174462	*Tc01v2_g019770* Ferrochelatase‐2, chloroplastic (21204419–21219868)	–	0.1364
	1	23747515	*Tc01v2_g020200* Uncharacterized protein (23778724–23781550)	–	0.0681
	1	31472241	*Tc01v2_g025400* Putative exopolygalacturonase clone GBGE184 (31467814–31469699)	–	0.0479
Branches with WBD	6	10714823	*Tc06v2_g005450* Putative NAC domain‐containing protein 35 (10785856–10788539)	Transcription regulation, DNA binding	0.1549
	1	32199021	*Tc01v2_g026530* RNA‐directed DNA polymerase (reverse transcriptase), ribonuclease H, putative (32199109–32200980)	–	0.1119
	9	1133400	*Tc09v2_g001870* Topless‐related protein 1 (1136104–1143084)	–	0.0899
	1	11181343	*Tc01v2_g014750* Carbohydrate‐binding X8 domain superfamily protein, putative (11171940–11173636)	–	0.0547
	4	6697943	*Tc04v2_g005150* Bifunctional protein FolD 1, mitochondrial (6714249–6720336)	Methenyltetrahydrofolate cyclohydrolase activity, methylenetetrahydrofolate dehydrogenase, tetrahydrofolate interconversion	0.0428

*Note*: SNPs are ranked by importance score (Portion). The table includes SNP identifier, chromosome (Chr), position, nearest gene (with genomic location), putative gene function, Gene Ontology (GO) annotation (if available), and importance score. Traits include healthy pod rate, pods with frosty pod rot (FPR), and flowers or branches with witches' broom disease (WBD). Genes in bold were also detected in the original GWAS conducted by Osorio‐Guarín et al.(2020) on the same dataset.

**TABLE 2:** Top single nucleotide polymorphisms (SNPs) from Bootstrap Forest and Boosted Tree genome‐wide association studies (GWAS) of cacao productivity and disease resistance using the Matina reference genome.

Trait	Scaffold	SNP position	Nearest genes and function	GO annotation	Importance score (Portion)
Bootstrap Forest					
Healthy pod rate	5	12534250	*Thecc1EG023287* PEBP (phosphatidylethanolamine‐binding protein) family protein (12598359–12601737)	–	0.0459
	3	3505073	*Thecc1EG012520* Uncharacterized protein DUF599 (3535034–3541320)	–	0.0348
	5	10101385	*Thecc1EG023050* RING/U‐box superfamily protein (10076217–10092402)	–	0.0225
	5	21539308	*Thecc1EG024023* Uncharacterized protein, chromo (CHRromatin Organisation MOdifier) domain containing (21306383–21649104)	–	0.0132
	5	11327144	*Thecc1EG023171* Uncharacterized protein (11271058–11294483)	–	0.0129
Pods with FPR	3	22872047	*Thecc1EG014511* Hydrolase, Galactinol–sucrose galactosyltransferase / raffinose synthase (22867979–22872961)	–	0.0615
	1	38575461	*Thecc1EG005862* Uncharacterized protein, PTHR12444//PTHR12444:SF0 ‐ uncharacterized // protein EFR3 homolog CMP44E (38570037–38577182)	–	0.0333
	1	18890898	*Thecc1EG003073* Uncharacterized protein (18887461–18892499)	–	0.0264
	1	27162270	*Thecc1EG003843* Gag‐protease‐integrase‐RT‐RNaseH polyprotein (27173246–27453604)	–	0.0262
	1	3947337	*Thecc1EG000880* Uncharacterized protein (3946411–3948266)	–	0.021
Flower with cushion WBD	2	794296	*Thecc1EG006100* Leucine‐rich repeat‐containing protein (794098–800964)	–	0.1478
	2	1107779	***Thecc1EG006180*** Uncharacterized protein, castor and pollux, part of voltage‐gated ion channel (1104002–1111419)	–	0.1402
	1	33051561	*Thecc1EG004701* Uncharacterized protein, reverse transcriptase‐like (RVT_3) // zinc‐binding in reverse transcriptase (zf‐RVT) (33049209–33052664)	–	0.0673
	2	4567399	***Thecc1EG006939*** Seven transmembrane MLO family protein (4565183–4572400)	–	0.0594
	7	2210089	*Thecc1EG030945* RNA‐binding CRS1 / YhbY domain‐containing protein (2204788–2210633)	–	0.043
Branches with WBD	9	1122735	*Thecc1EG036963* TOPLESS‐related 1 (1118542–1132915)	–	0.0355
	6	8932900	*Thecc1EG027932* Uncharacterized protein (8920672–8943551)	–	0.0325
	10	2638347	*Thecc1EG042851* Uncharacterized protein (2585353–2812295)	–	0.0285
	7	3661030	*Thecc1EG046864* S‐locus lectin protein kinase family protein (3663242–3667965)	–	0.0254
	9	20317281	*Thecc1EG039538* Uncharacterized protein (20318482–20318929)	–	0.0206
Boosted Tree					
Healthy pod rate	5	11327144	*Thecc1EG023171* Uncharacterized protein (11271058–11294483)	–	0.1182
	5	10101385	*Thecc1EG023050* RING/U‐box superfamily protein (10076217–10092402)	–	0.1006
	3	3505073	*Thecc1EG012520* Uncharacterized protein DUF599 (3535034–3541320)	–	0.0968
	1	7438454	*Thecc1EG001487* Ankyrin repeat‐containing protein (7436699–7439430)	–	0.0673
	4	8343325	*Thecc1EG017880* Uncharacterized protein, PF00385 ‐ Chromo (CHRromatin Organisation MOdifier) domain (Chromo) (8340704–9011110)	–	0.0288
Pods with FPR	6	7186038	*Thecc1EG027776* Uncharacterized protein, troponin coil‐coiled subunits containing (7189328–7189674)	–	0.1886
	3	22872047	*Thecc1EG014511* Hydrolase, Galactinol–sucrose galactosyltransferase / raffinose synthase (22867979–22872961)	–	0.1796
	8	9466348	*Thecc1EG035323* Dead box ATP‐dependent RNA helicase (8092198–10317647)	–	0.0471
	3	4938189	*Thecc1EG012671* 21 kDa seed protein, trypsin and protease inhibitor (4976844–4993230)		0.0326
	5	23097582	*Thecc1EG024191* DegP protease 10, serine protease family S1C HTRA‐related (23061719–23081514)	–	0.0321
Flower with cushion WBD	1	33051561	*Thecc1EG004701* Uncharacterized protein, reverse transcriptase‐like (RVT_3) // zinc‐binding in reverse transcriptase (zf‐RVT) (33049209–33052664)	–	0.2189
	2	1107779	***Thecc1EG006180*** Uncharacterized protein, castor and pollux, part of voltage‐gated ion channel (1104002–1111419)	–	0.2071
	2	794296	*Thecc1EG006100* Leucine‐rich repeat‐containing protein (794098–800964)	–	0.127
	5	6787809	*Thecc1EG022711* Uncharacterized protein (6796784–6802454)	–	0.0804
	4	23054194	*Thecc1EG019515* Retrotransposon gag protein (23051547–23053027)	–	0.0452
Branches with WBD	9	28258940	*Thecc1EG040241* RNA‐dependent RNA polymerase 2 (28257011–28264572)	–	0.1129
	6	8932900	*Thecc1EG027932* Uncharacterized protein (8920672–8943551)	–	0.1047
	9	1122735	*Thecc1EG036963* TOPLESS‐related 1 (1118542–1132915)	–	0.0962
	5	12670234	*Thecc1EG023288* Uncharacterized protein (2604989–12617304)		0.0907
	9	20317281	*Thecc1EG039538* Uncharacterized protein (20318482–20318929)	–	0.0631

*Note*: The table includes SNP identifier, scaffold, position, nearest gene (with genomic location), putative gene function, and importance score. Traits analyzed include healthy pod rate, pods with FPR, flowers with cushion WBD, and branches with WBD. GO annotation was not available for any of the listed genes. Genes in bold were also detected in the original GWAS conducted by Osorio‐Guarín et al.(2020) on the same dataset.

**TABLE 3:** Concordant candidate gene associations for cacao disease resistance and productivity traits identified by both ML genome‐wide association studies (GWAS) (current study) and traditional GWAS (Osorio‐Guarín et al., 2020).

Trait	Chr	SNP position	Nearest genes and function	GO annotation	Reference genome	Model
Flower with cushion WBD All traits combined PC1	2	1064432	*Tc02v2_g001650* Ion channel DMI1 (1060675–1066653)	–	Criollo	BT, BF
Flower with cushion WBD All traits combined PC1	2	770436	*Tc02v2_g001090* Probable xyloglucan galactosyltransferase GT17 (768191–770560)	–	Criollo	BT, BF
Flower with cushion WBD All traits combined	2	4523324	*Tc02v2_g007330* MLO‐like protein 4 (4521274–4527984)	–	Criollo	BF
Flower with cushion WBD All traits combined PC1	2	1107779	*Thecc1EG006180* Uncharacterized protein, castor and pollux, part of voltage‐gated ion channel (1104002–1111419)	–	Matina	BT, BF
Flower with cushion WBD All traits combined	2	4567399	*Thecc1EG006939* Seven transmembrane MLO family protein (4565183–4572400)	–	Matina	BT, BF
Pods with FPR	8	9466348	*Thecc1EG035323* Dead box ATP‐dependent RNA helicase (8092198–10317647)	–	Matina	BT

Abbreviations: BF, Bootstrap Forest; BT, Boosted Tree; FPR, frosty pod rot; ML, machine learning; PC1, first principal component; WBD, witches' broom disease.

To evaluate the potential predictive performance and stability of different modeling approaches given the dataset characteristics (102 accessions, > 8000 SNPs), a preliminary *K* = 5 cross‐validation was performed using the “Model Screening” platform in JMP Pro 17. This analysis compared K‐nearest neighbors, BF, support vector machines (RBF kernel), BT, and Decision Tree algorithms using default settings. The results indicated that while BF and BT models often achieved high explanatory power on the training sets (average training R‐squared across the 5 folds frequently exceeding 0.80 and sometimes over 0.90, depending on the trait and reference genome, e.g., 0.88 for BF and 0.93 for BT for Flower Cushion WBD), performance on the corresponding validation sets was highly variable and generally low (validation R‐squared values ranging from negative values up to over 0.5 [Decision tree and BF for flower with cushion WBD in Matina genome], but mostly around 0.20 or less). This instability in validation performance was attributed to the limited sample size relative to the number of predictors, making robust estimation on hold‐out data unreliable for assessing predictive accuracy or SNP importance. Therefore, to maximize the stability of SNP importance estimation for identifying potentially relevant loci within this specific dataset, the final BF and BT models used for generating the importance rankings reported in this study were trained using the entire available dataset.

To investigate the combined genetic effects on the four traits, we conducted two additional GWAS analyses using composite phenotypic measures derived from the original traits. First, we created a new variable representing the product of the four traits for each accession, calculated by multiplying the values of healthy pod rate, pods with FPR, flower cushions with WBD, and branches with WBD. Second, we performed a principal component analysis (PCA) on the four traits using JMP Pro 17 to extract the first principal component (PC1). As PC1 captures the largest proportion of shared variance among the measured traits, we utilized it as a composite phenotype in an exploratory GWAS aimed at identifying loci associated with the primary axis of phenotypic variation, potentially reflecting overall plant performance across these productivity and disease resistance metrics or underlying pleiotropic genetic effects. Subsequently, GWAS was carried out using both the product variable and PC1 as the response variables, following the BF and BT procedures, utilizing both the Criollo (https://cocoa‐genome‐hub.southgreen.fr/) and Matina (https://phytozome‐next.jgi.doe.gov/info/Tcacao_v1_1) datasets. Gene annotations, including Gene Ontology (GO) terms and putative functions, were obtained from UniProt Knowledgebase (UniProtKB; https://www.uniprot.org/).

### 2.5 Correlation analysis of SNP importance scores

To assess the consistency of SNP associations across different traits and ML models, we performed a correlation analysis using the SNP importance scores derived from the GWAS. For each reference genome (Criollo and Matina), we extracted the importance score (Portion) for each SNP from each of the eight analyses (4 traits × 2 models). This resulted in a matrix of SNP importance scores, where each row represented an SNP and each column represented a specific trait and model combination (e.g., Healthy pod rate—BF, Pods with FPR—BT).

Pearson's correlation coefficients were then calculated between all pairs of columns in the importance score matrix using the “Multivariate” platform within JMP Pro 17. The resulting correlation coefficients, which range from −1 to +1, reflect the degree and direction of the linear relationship between the SNP importance scores for different traits and models. A positive correlation indicates that SNPs with high importance for one trait/model tend to have high importance for the other trait/model, while a negative correlation indicates the opposite.

## 3 RESULTS

### 3.1 Weak correlations between genetic distance and phenotypic traits in cacao

**FIGURE 1:**
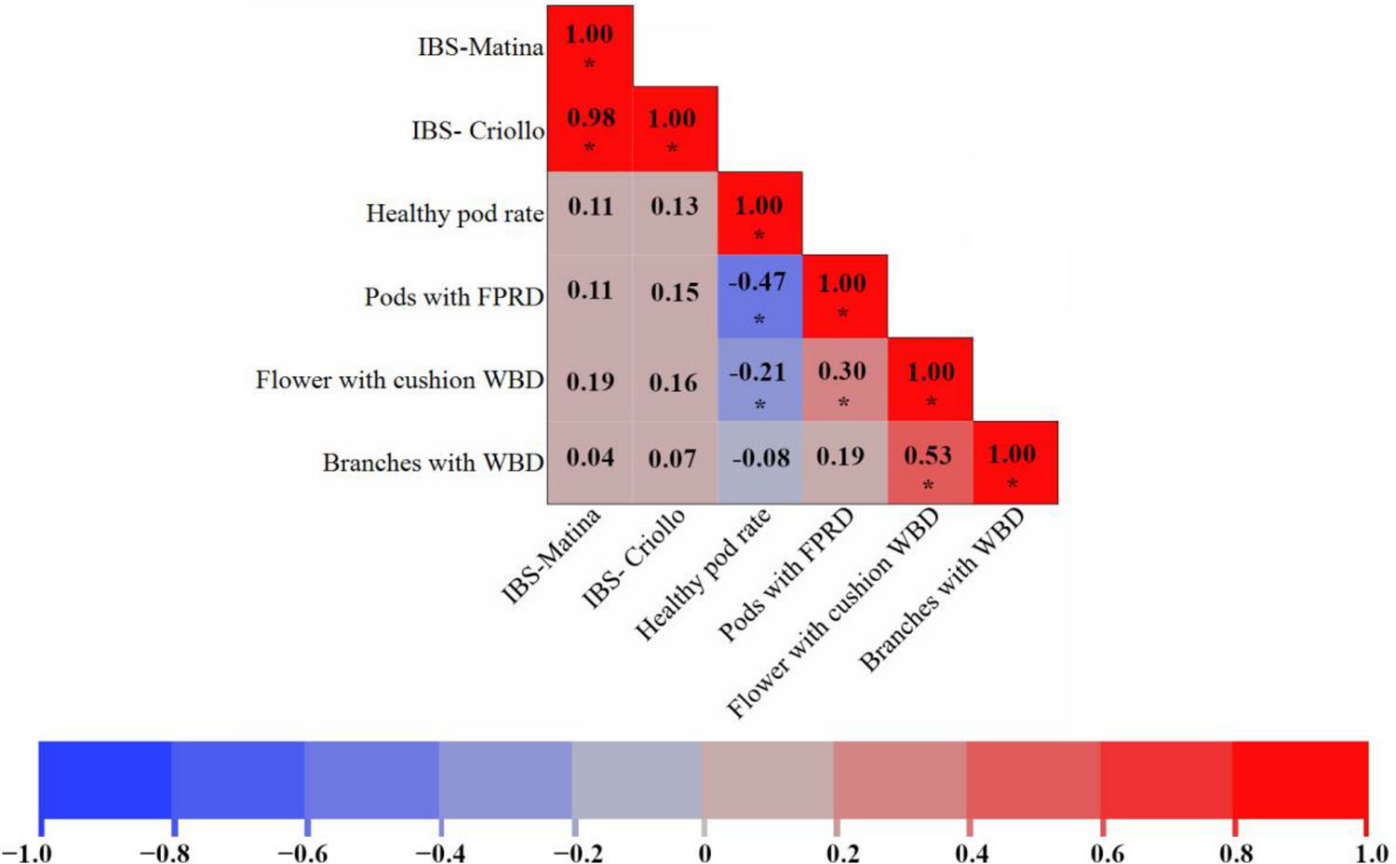
Correlation between genetic distance and phenotypic traits in cacao. Heatmap of Pearson's correlation coefficients between genetic distances and four phenotypic traits in cacao. Genetic distances were calculated using the IBS method based on SNP markers mapped to the Matina (IBS‐Matina) and Criollo (IBS‐Criollo) genomes. Phenotypic traits include healthy pod rate, AUDPC for pods with FPR, AUDPC for flower cushions with WBD, and AUDPC for branches with WBD. Red indicates a positive correlation, blue indicates a negative correlation, and color intensity represents the magnitude of the correlation. Asterisks (*) denote statistically significant correlations (*p* < 0.05). FPRD, frosty pod rot disease; IBS, identity by state; WBD, witches' broom disease.

To investigate the relationship between genetic relatedness and phenotypic variation in our cacao collection, we calculated Pearson's correlation coefficients between genetic distances and four key traits: healthy pod rate, pods with FPR, flower cushions with WBD, and branches with WBD (Figure 1). Genetic distances were computed using the IBS method based on SNP markers mapped to two different reference genomes, Matina (IBS‐Matina) and Criollo (IBS‐Criollo). Overall, the correlations between genetic distance and the four traits were generally weak (Figure 1). The two genetic distance measures, IBS‐Matina and IBS‐Criollo, were highly correlated with each other (*r* = 0.98, *p* < 0.0001), indicating that the choice of reference genome had a minimal impact on the overall assessment of genetic relationships among these accessions. Healthy pod rate showed weak, nonsignificant positive correlations with both IBS‐Matina (*r* = 0.11, *p* > 0.05) and IBS‐Criollo (*r* = 0.13, *p* > 0.05). This suggests that genetic similarity, as measured by IBS, is not a strong predictor of healthy pod production in this collection.

For disease resistance traits, the correlations were also generally weak. Pods with FPR exhibited a weak, nonsignificant positive correlation with IBS‐Matina (*r *= 0.11, *p* > 0.05) and a weak, nonsignificant positive correlation with IBS‐Criollo (*r* = 0.15, *p *> 0.05). A statistically significant (*p* < 0.05) moderate negative correlation (*r* = ‐0.47) was observed between healthy pod rate and pods with FPR, indicating that accessions with higher healthy pod rates tended to have lower susceptibility to FPR as described by Osorio‐Guarín et al.(2020). Similarly, a significant positive correlation was observed between flower cushions with WBD and branches with WBD (*r* = 0.53, *p* < 0.05), as expected since they are both measures of susceptibility to the same disease.

### 3.2 Genetic structure and trait associations in cacao accessions

**FIGURE 2:**
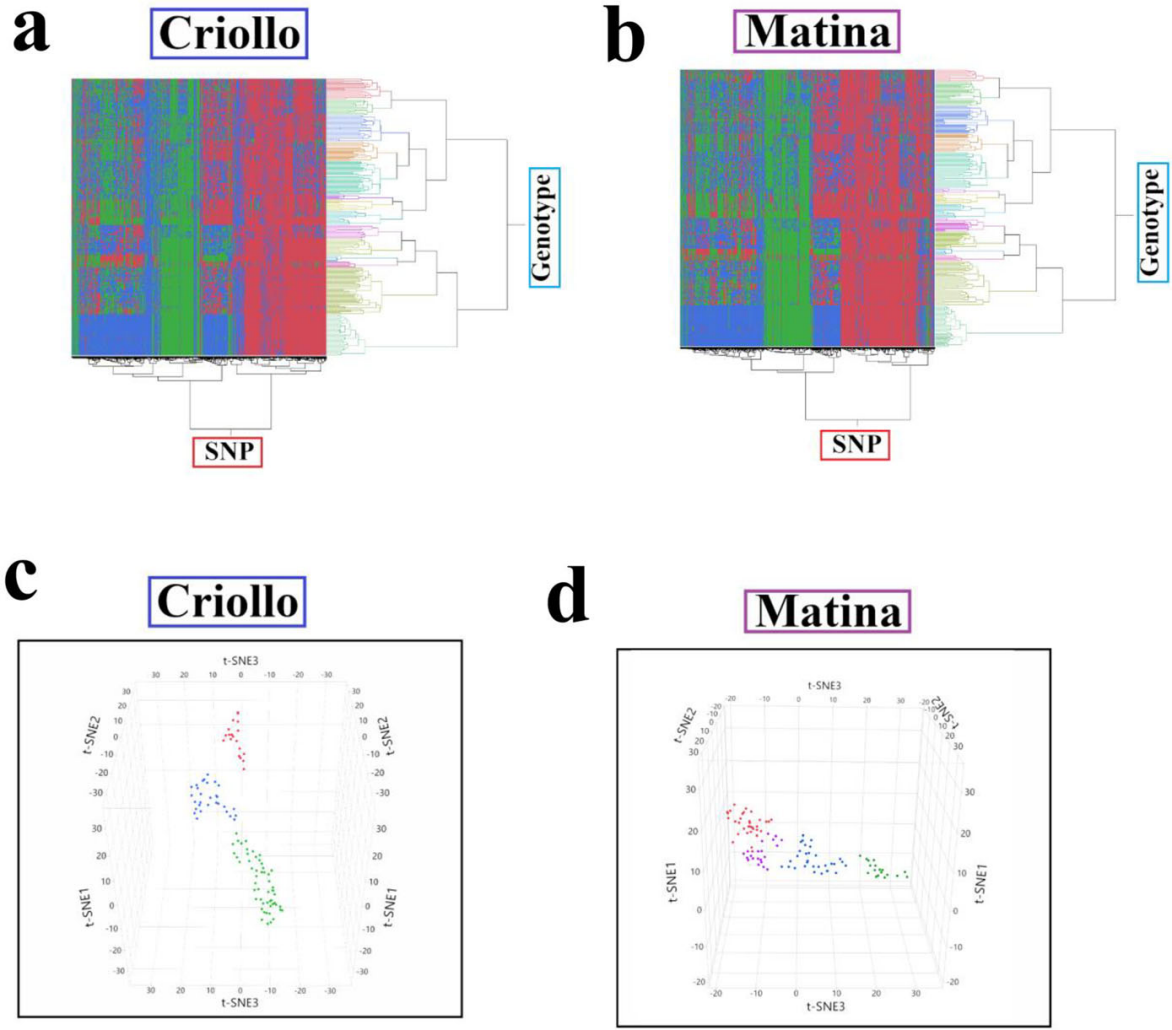
Genomic and phenotypic diversity in cacao. Visualization of genetic and phenotypic diversity among 229 cacao accessions. (a and b) Hierarchical clustering based on single nucleotide polymorphism (SNP) data mapped to either the (a) Criollo or (b) Matina reference genomes. (c and d) t‐distributed stochastic neighbor embedding (t‐SNE) plots based on SNP and four phenotype data for (c) Criollo and (d) Matina, revealing three or four primary clusters within the collection.

Hierarchical clustering based on SNP data revealed complex relationships among the accessions, with no single clustering solution perfectly capturing the diversity within the collection (Figure 2a,b). The dendrograms for both Criollo and Matina showed that the accessions formed 14 distinct clusters, as determined by considering the underlying genetic structure. However, several noteworthy patterns emerged. The heatmaps, representing the SNP genotypes, visually illustrated the genetic similarities and differences among accessions and clusters. While some clusters appeared relatively homogeneous in terms of their SNP profiles, others exhibited greater within‐cluster diversity. The specific patterns of clustering and the composition of the clusters differed somewhat between the analyses based on the Criollo and Matina genomes, suggesting that the choice of reference genome can exert a slight influence on the resolution of population structure, although there was clear congruence between the two analyses, showing nearly identical heatmaps.

Complementing the hierarchical clustering, t‐SNE, a nonlinear dimensionality reduction technique, provided an alternative visualization of the genetic structure, incorporating both SNP and phenotypic data (Figure 2c,d). When applied to the combined dataset, t‐SNE generated two‐dimensional plots that revealed distinct groupings for both Criollo and Matina. The t‐SNE plots effectively captured the relationships between genetic variation and phenotypic traits, with clusters appearing relatively well‐separated in the reduced‐dimensional space. Notably, the t‐SNE plots also revealed a degree of overlap between the clusters, particularly in the Matina‐based analysis, suggesting the presence of admixture or intermediate genotypes, consistent with the known complex history of cacao domestication and breeding. The specific arrangements of accessions within and between clusters differed somewhat between the Criollo and Matina‐based t‐SNE analyses, further emphasizing the influence of the reference genome on the resolution of population structure and trait associations.

### 3.3 Identification of candidate genes associated with disease resistance through BF and BT‐based GWAS in the Criollo genome

To pinpoint genomic regions associated with cacao productivity (healthy pod rate) and disease resistance, we conducted a GWAS using both BF and BT models. We analyzed four key traits: healthy pod rate, pods with FPR, flowers with cushion WBD, and branches with WBD, using SNP markers mapped to the Criollo reference genome.

**FIGURE 3:**
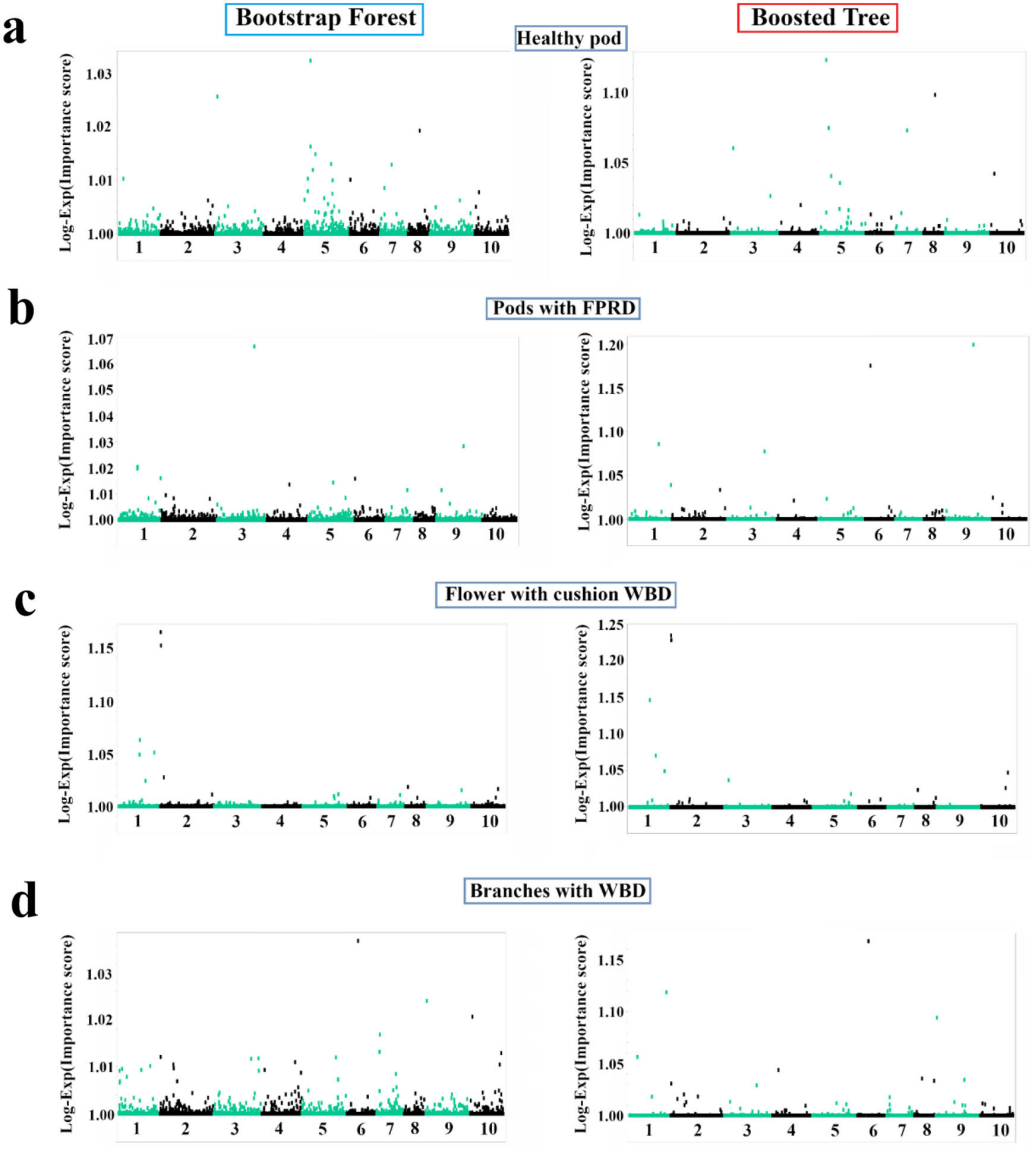
Genome‐wide association study of cacao disease resistance and healthy pod rate using single nucleotide polymorphism (SNP) markers mapped to the Criollo genome. Manhattan plots showing the results of a genome‐wide association studies (GWAS) for four traits in cacao using SNP markers mapped to the Criollo reference genome with Bootstrap Forest (left) and Boosted Tree (right) models. (a) Healthy pod rate, (b) pods with frosty pod rot (FPR), (c) flower with cushion WBD, and (d) branches with witches' broom disease (WBD). The *x*‐axis represents the chromosomal location of each SNP along the 10 cacao chromosomes. The *y*‐axis represents the Log‐Exp(Importance score) for each SNP, a measure of its association with the trait.

Our analyses identified numerous SNPs significantly associated with productivity and disease resistance traits in cacao. These associations, detailed in Table 1 and visualized in Figure 3, highlight a diverse set of candidate genes identified by both the BF and BT models. These genes are implicated in a range of biological processes, notably cell wall biosynthesis and modification, response to biotic and abiotic stress, and defense‐related mechanisms, which include potential roles in RNA silencing and ion transport. For example, we found strong associations with genes encoding a putative xyloglucan galactosyltransferase, a DEAD‐box ATP‐dependent RNA helicase and an ion channel protein (Table 1).

### 3.4 Identification of candidate genes associated with productivity and disease resistance through BF and BT‐based GWAS in the Matina genome

**FIGURE 4:**
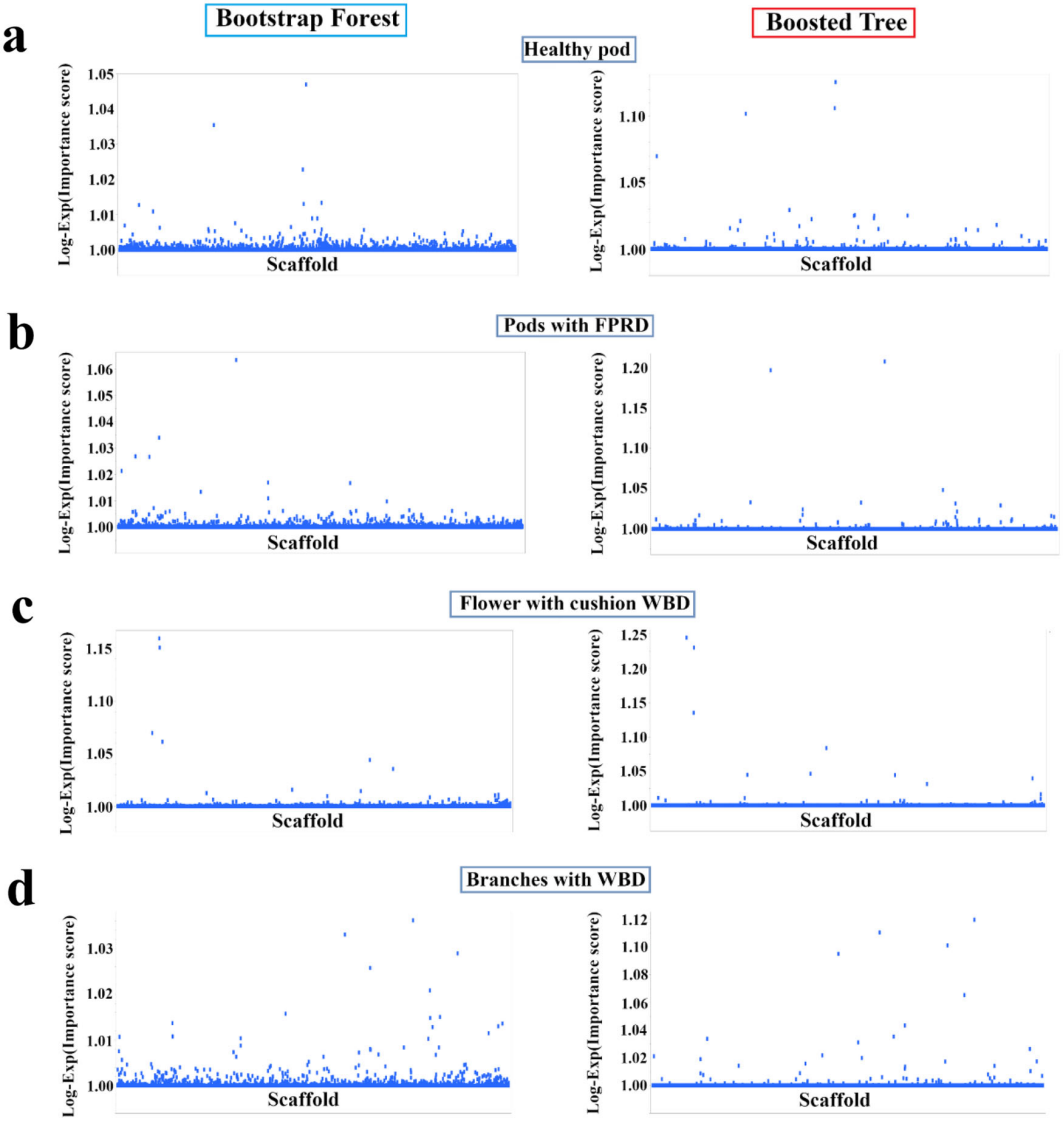
Manhattan plots from genome‐wide association studies (GWAS) of productivity and disease resistance in cacao using the Matina reference genome. The results are shown for four traits: (a) healthy pod rate, (b) pods with frosty pod rot (FPR), (c) flower cushions with WBD, and (d) branches with witches' broom disease (WBD). Bootstrap Forest (left) and Boosted Tree (right) models were used to assess single nucleotide polymorphism (SNP)–trait associations. The *x*‐axis represents the physical position of each SNP along the 10 cacao chromosomes/scaffolds. The *y*‐axis displays the Log‐Exp(Importance score), reflecting the strength of its association with the trait.

To further investigate the genetic basis of cacao pod health and disease resistance, we conducted additional GWAS using SNP markers mapped to the Matina reference genome. Again, both BF and BT models were used to analyze the same four key traits: healthy pod rate, pods with FPR, flower cushions with WBD, and branches with WBD. Our analyses revealed several SNPs with significant associations with these traits in the Matina genome. The top SNPs and their associated candidate genes, identified using both models, are detailed in Table 2 and visualized in Figure 4. Similar to the Criollo genome analysis, we found associations with genes implicated in diverse biological processes, including potential roles in cell wall modification, stress response, and defense mechanisms. We identified candidate genes with putative functions in defense response, such as *Thecc1EG006100* encoding a Leucine‐rich repeat‐containing protein and *Thecc1EG040241* encoding an RNA polymerase 2, as well as *Thecc1EG036963*, a TOPLESS‐related 1 protein involved in transcriptional regulation. Notably, the specific genes and genomic regions associated with each trait differed between the Matina and Criollo analyses, indicating the influence of the reference genome on GWAS results and the presence of a complex genetic architecture underlying these traits.

### 3.5 Analysis of combined traits using multiplicative index and principal component analysis

**FIGURE 5:**
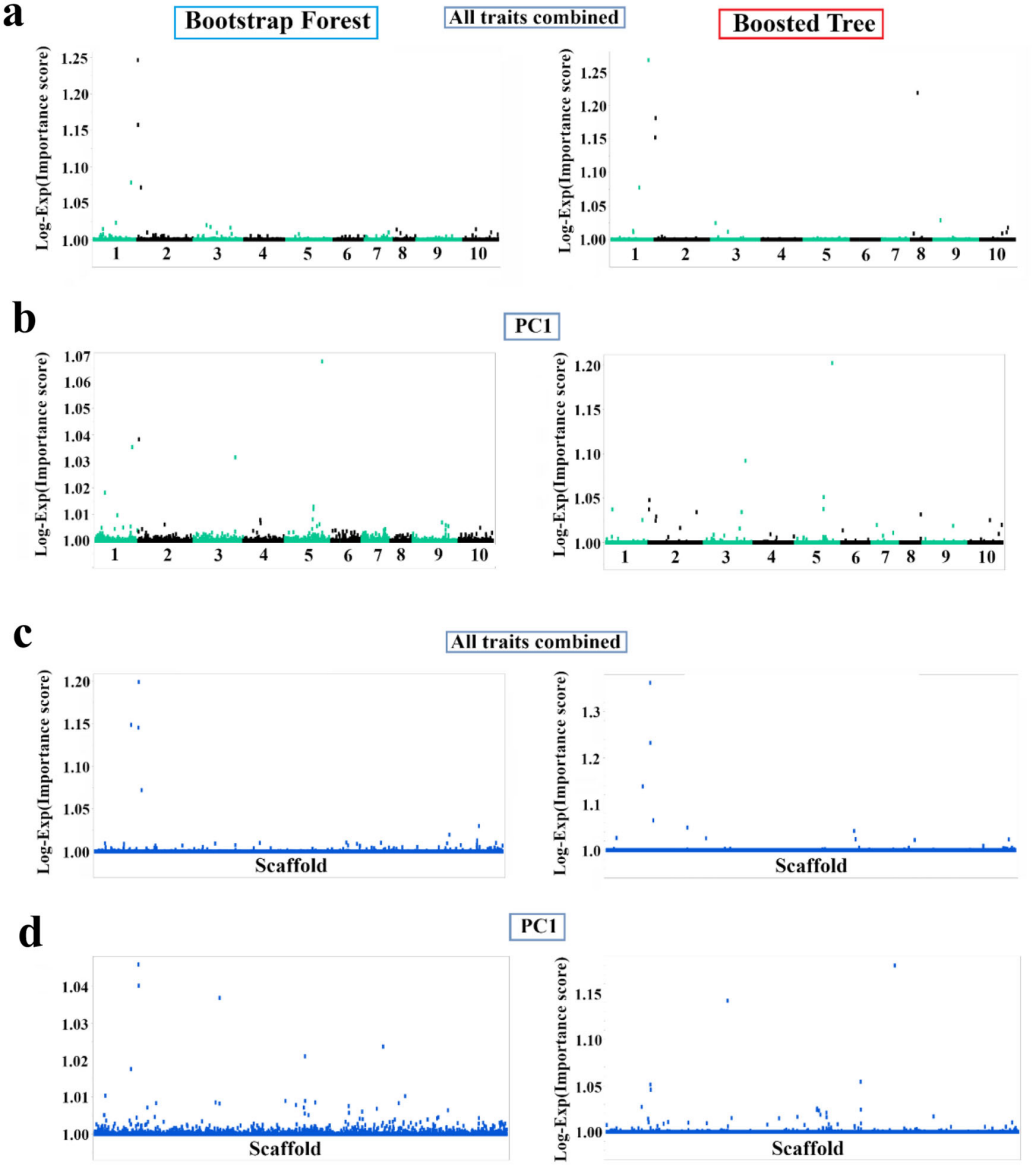
Genome‐wide association studies (GWAS) of combined cacao traits using a multiplicative index and first principal component (PC1). Manhattan plots show results for (a) multiplicative index of traits using the Criollo genome, (b) PC1 using the Criollo genome, (c) multiplicative index of traits using the Matina genome, And (d) PC1 using the Matina genome. *X*‐axis: physical position of each single nucleotide polymorphism (SNP) along the 10 cacao chromosomes/scaffolds. *Y*‐axis: Log‐Exp(Importance score).

To explore the genetic architecture of cacao productivity and disease resistance through a combined trait approach, we performed GWAS using two composite measures: a multiplicative index of four traits and their first principal component. These analyses were conducted using both BF and BT models, with SNP markers mapped to either the Criollo or Matina reference genomes. The results, detailed in Table S1 and visualized in Figure 5, revealed several significant SNP associations with both composite measures. When using the Criollo genome, several SNPs were found to be associated with both the multiplicative index and PC1, including SNPs near genes encoding for a probable xyloglucan galactosyltransferase GT17 (*Tc02v2_g001090*) and an ion channel DMI1 (*Tc02v2_g001650*). For the Matina genome, we identified associated SNPs near genes encoding for a Leucine‐rich repeat‐containing protein (*Thecc1EG006100*) and an uncharacterized protein with a castor and pollux, part of voltage‐gated ion channel (*Thecc1EG006180*). The specific SNPs and candidate genes identified differed depending on the composite measure used and the reference genome, corroborating our other findings that confirm the complexity of these traits and the influence of the reference genome on GWAS results.

**FIGURE 6:**
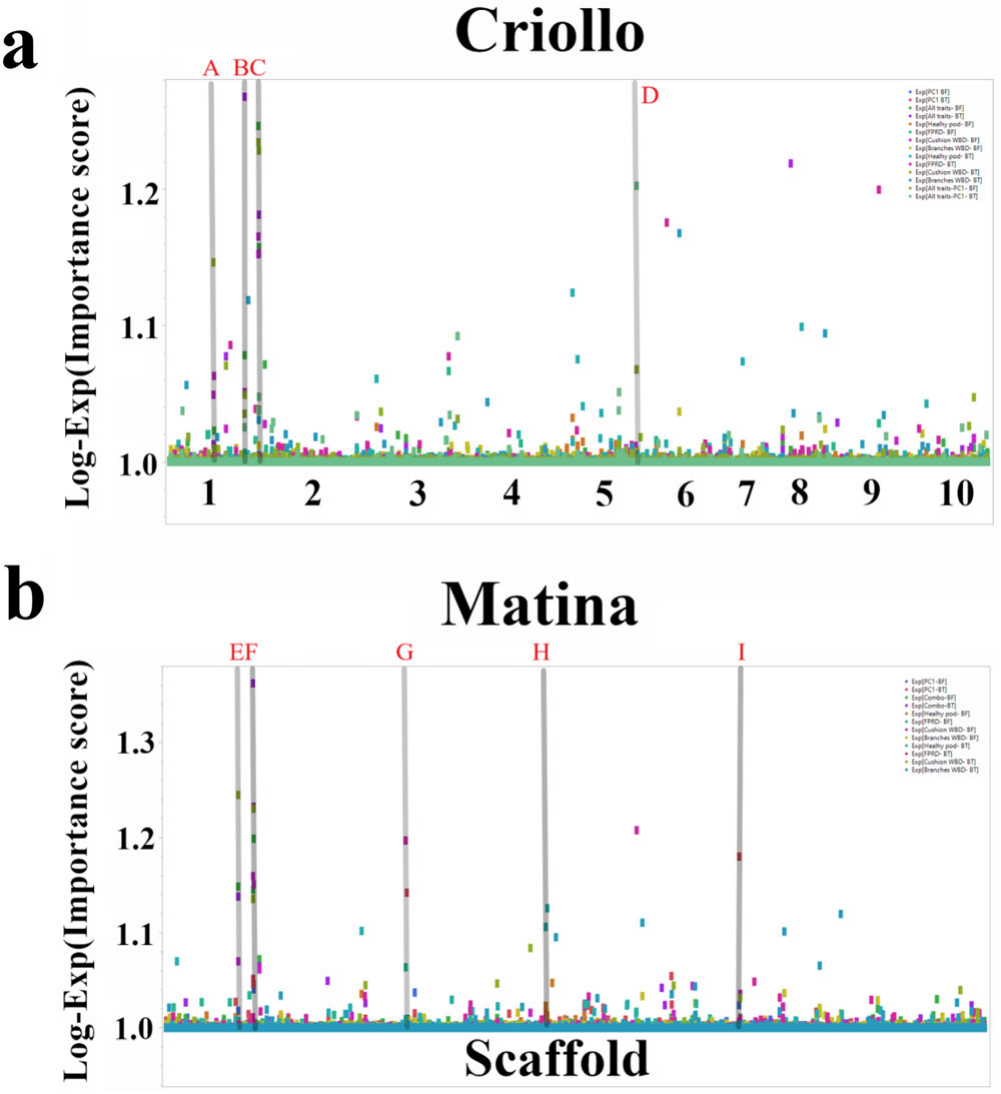
Integrative genome‐wide association studies (GWAS) of cacao productivity and disease resistance using Bootstrap Forest and Boosted Tree models and two reference genomes. Manhattan plots show combined association results for healthy pod rate and resistance to three diseases: pods with frosty pod rot (FPR), flower cushions with witches' broom disease (WBD), and branches with WBD. (a) Criollo genome. (b) Matina genome. Each point represents a single nucleotide polymorphism (SNP). X‐axis: physical position along the ten chromosomes/scaffolds. *Y*‐axis: Log‐Exp(Importance score), a combined measure of association strength from both models. Highlighted peaks (A–I) correspond to notable SNP–trait associations and their potential candidate genes: A: Chr1_21174462 (*Tc01v2_g019770*, ferrochelatase‐2, chloroplastic); B: Chr1_31472241 (*Tc01v2_g025400*, putative exopolygalacturonase); C: Chr2_770436 (*Tc02v2_g001090*, probable xyloglucan galactosyltransferase GT17); D: Chr5_29556644 (*Tc05v2_g015380*, flavonol sulfotransferase‐like protein); E: Scaffold_1_33051561 (*Thecc1EG004701*, uncharacterized protein with reverse transcriptase‐like and zinc‐binding domains); F: Scaffold_2_794296 (*Thecc1EG006100*, leucine‐rich repeat‐containing protein); G: Scaffold_3_22872047 (*Thecc1EG014511*, galactinol‐sucrose galactosyltransferase/raffinose synthase); H: Scaffold_5_11327144 (*Thecc1EG023171*, uncharacterized protein); I: Scaffold_8_2342743 (*Thecc1EG034244*, clathrin adaptor complex medium subunit family protein).

To comprehensively visualize the genomic regions associated with cacao productivity and disease resistance, we integrated the results of our GWAS across all four traits, incorporating both ML models as well as both reference genomes, and displayed them in Manhattan plots (Figure 6). This combined visualization revealed several prominent peaks, representing SNPs with strong composite association signals across traits, models, and genomes. Notably, several candidate genes identified in our ML‐based analyses were also reported in the original study by Osorio‐Guarín et al. (2020) using traditional GWAS methods, highlighting concordance between the approaches; these overlapping findings are summarized in Table 3. In the Criollo analysis, prominent peaks were located on chromosome 1 near genes encoding ferrochelatase‐2 and a putative exopolygalacturonase (Figure 6a, peaks A and B), on chromosome 2 near a gene encoding a probable xyloglucan galactosyltransferase GT17 (Figure 6a, peak C), and on chromosome 5 near a gene encoding a flavonol sulfotransferase‐like protein (Figure 6a, peak D). In the Matina analysis, notable peaks were found on several scaffolds, including those near genes encoding a leucine‐rich repeat‐containing protein and a galactinol‐sucrose galactosyltransferase/raffinose synthase (Figure 6b, peaks F and G). These findings highlight the potential involvement of diverse biological processes (e.g., iron metabolism, cell wall modification, carbohydrate metabolism, stress response) in determining the overall well‐being and performance of cacao plants, ultimately influencing productivity and disease resistance. A complete list of highlighted peaks and their associated candidate genes is provided in the caption of Figure 6.

### 3.6 Correlation of SNP importance scores across traits and models

**FIGURE 7:**
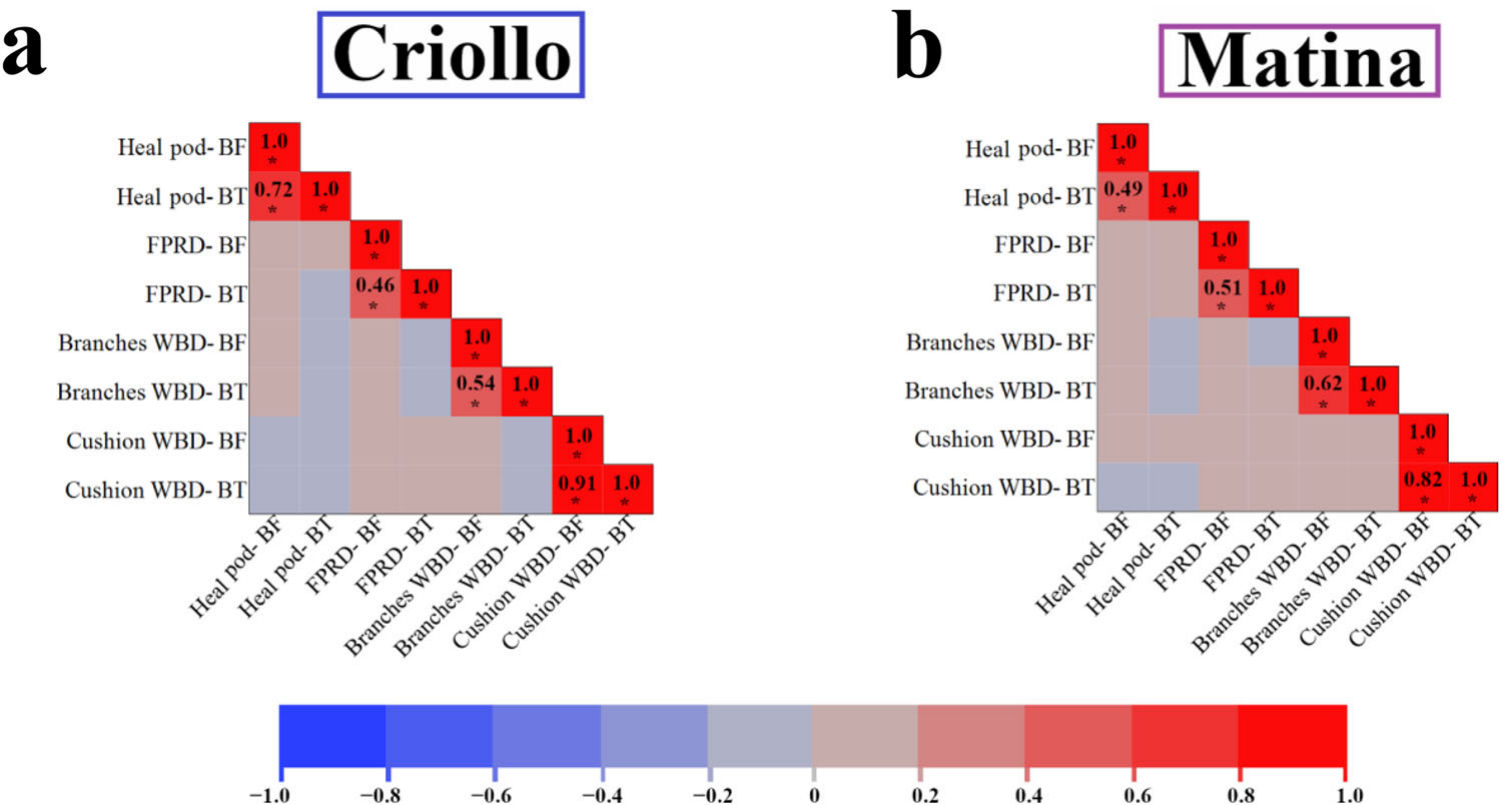
Consistency of single nucleotide polymorphism (SNP) importance scores across traits and models in cacao genome‐wide association studies (GWAS). Heatmaps show Pearson's correlations between SNP importance scores from Bootstrap Forest (BF) and Boosted Tree (BT) models for four traits: healthy pod rate, pods with frosty pod rot (FPR), flower cushions with witches' broom disease (WBD), and branches with WBD. (a) Criollo genome. (b) Matina genome. Cell color intensity indicates correlation strength (red: positive, blue: negative); asterisks (*) denote significance (*p* < 0.05). Only significant correlation coefficients considered to be practically meaningful (*r* > 0.1) are displayed numerically.

To assess the consistency of SNP associations across different traits and ML models, we calculated Pearson's correlation coefficients between SNP importance scores derived from the GWAS analyses (Figure 7). These analyses were performed separately using SNP data mapped to the Criollo (Figure 7a) and Matina (Figure 7b) reference genomes. In the Criollo genome analysis, the highest correlation (*r* = 0.91, *p* < 0.0001) was observed between the two WBD traits (Cushion WBD‐BF and Cushion WBD‐BT), indicating strong agreement between models for this trait (Figure 7a). Similarly, in the Matina genome analysis, the two Cushion WBD traits also showed the highest correlation (*r* = 0.82, *p* < 0.05) (Figure 7b). For both the Criollo and Matina genomes, correlations between other trait–model combinations were generally weaker or nonsignificant, suggesting that different sets of SNPs may influence each trait, and that model choice can impact the identification of these associations. However, in the Matina genome, a moderate positive correlation was observed between the two models for FPR (*r *= 0.51, *p* < 0.05) (Figure 7b). The overall pattern of correlations suggests that while there is some consistency in SNP importance across models for certain traits, particularly WBD, the genetic architecture of these traits is complex and may involve distinct sets of genes.

### 3.7 Overlap of top SNP associations between models and traits

**FIGURE 8:**
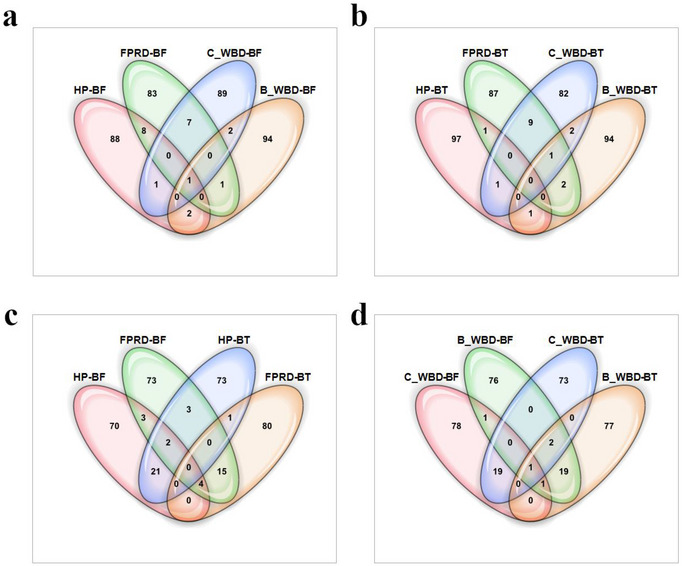
Venn diagrams illustrating the overlap among the top single nucleotide polymorphisms (SNPs) identified by Bootstrap Forest (BF) and Boosted Tree (BT) models for cacao disease resistance and productivity traits using the Criollo reference genome. Sets represent the up to top 100 SNPs based on importance score for: healthy pod rate (HP), frosty pod rot disease (FPRD), flower cushion witches' broom disease (C_WBD), and branch witches' broom disease (B_WBD) (note: in cases where fewer than 100 SNPs had a non‐zero importance score for a given trait/model combination, the set includes all SNPs with a non‐zero score). Overlapping regions indicate SNPs identified as important for multiple traits and/or by different models. (a) Overlap among the top SNPs for the four traits identified using the BF model. (b) Overlap among the top SNPs for the four traits identified using the BT model. (c) Overlap among the top SNPs for selected trait‐model combinations: HP‐BF, FPRD‐BF, FPRD‐BT, and C_WBD‐BT. (d) Overlap among the top SNPs for WBD traits (C_WBD and B_WBD) identified by both BF and BT models.

**FIGURE 9:**
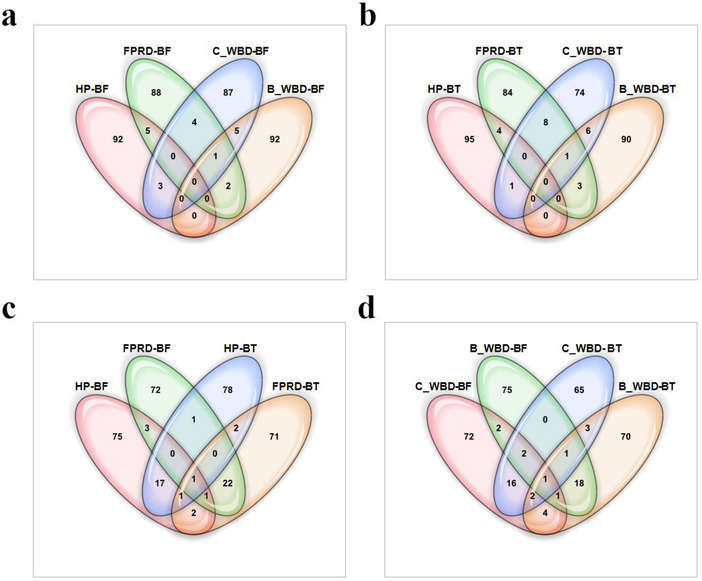
Venn diagrams illustrating the overlap among the top single nucleotide polymorphisms (SNPs) identified by Bootstrap Forest (BF) and Boosted Tree (BT) models for cacao disease resistance and productivity traits using the Matina reference genome. Sets represent the up to top 100 SNPs based on importance score for: healthy pod rate (HP), frosty pod rot disease (FPRD), flower cushion witches' broom disease (C_WBD), and branch witches' broom disease (B_WBD) (note: in cases where fewer than 100 SNPs had a non‐zero importance score for a given trait/model combination, the set includes all SNPs with a non‐zero score). Overlapping regions indicate SNPs identified as important for multiple traits and/or by different models. (a) Overlap among the top SNPs for the four traits identified using the BF model. (b) Overlap among the top SNPs for the four traits identified using the BT model. (c) Overlap among the top SNPs for HP and FPRD traits identified by both BF and BT models. (d) Overlap among the top SNPs for WBD traits (C_WBD and B_WBD) identified by both BF and BT models.

To further investigate the relationships between the top SNPs identified by the different models and for different traits, the overlap among the top SNPs (up to 100 with non‐zero importance scores) was visualized using Venn diagrams for the Criollo (Figure 8) and Matina (Figure 9) reference genomes.

Consistent with the correlation analysis (Figure 7), the diagrams generally show limited overlap between the top SNPs identified for distinct traits, particularly when considering SNPs found uniquely by only one model (Figures 8a,b and 9a,b). For instance, using the BF model on the Criollo genome, only one SNP (chr5_29556644, near the flavonol sulfotransferase‐like gene *Tc05v2_g015380*) was found among the top SNPs for all four traits simultaneously (Figure 8a), suggesting that largely different sets of SNPs are prioritized for each specific trait.

However, comparing the findings for the same trait across the two different models (BF vs. BT) reveals greater concordance for some traits, especially WBD. For Flower Cushion WBD (C_WBD), 19 top SNPs were common to both BF and BT models in the Criollo analysis (Figure 8d) and 16 SNPs were common in the Matina analysis (Figure 9d). Similarly, for Branch WBD (B_WBD), 19 SNPs were shared between models for Criollo (Figure 8d) and 18 for Matina (Figure 9d). This concordance lends further confidence to these shared associations. Notably, the single SNP (chr5_29556644) identified as important for all four traits by the BF model in Criollo was also identified by both models for both WBD traits (central overlap in Figure 8d). Similarly, in the Matina analysis, one SNP (SCAFFOLD_2_9415422, near a protein kinase/lectin gene *Thecc1EG007853*) was identified by both models for both WBD traits (central overlap in Figure 9d), indicating these may represent particularly robust candidate loci for broad WBD responses.

Overlap between models for Healthy Pod rate and frosty pod rot disease (FPRD) traits appeared less pronounced overall in the top SNP sets visualized, although some robust SNPs were identified, such as SCAFFOLD_3_22872047 (near the galactinol‐sucrose synthase gene *Thecc1EG014511*), which was found in the top SNPs for both HP and FPRD by both BF and BT models in the Matina analysis (central overlap in Figure 9c).

## 4 DISCUSSION

This study utilized a ML‐based GWAS to investigate the complex genetic architecture of productivity and disease resistance in a diverse cacao collection. We identified numerous candidate genes associated with healthy pod rate, resistance to FPR, and resistance to WBD (Figures 3, 4, 5, 6, Tables 1, 2, 3, and Table S1), revealing a complex interplay of loci influencing these traits. Analysis of population structure using hierarchical clustering and t‐SNE (Figure 2) indicated a high degree of admixture within the collection, reflecting cacao's history of domestication and breeding (Argout et al., 2011). The observed weak correlations between genetic distance, as measured by IBS, and the phenotypic traits examined suggest that overall genetic similarity is not a strong predictor of productivity or disease resistance in this cacao collection. This finding, consistent with findings in other complex traits in plants (Lindhout, 2002), implies that these traits are likely governed by a complex polygenic architecture involving numerous genes, each with a relatively small effect. The high degree of admixture suggested by the t‐SNE analysis (Figure 2), which revealed overlapping groupings rather than distinct clusters, likely contributes to these weak correlations. This admixture reflects the complex history of cacao domestication, hybridization, and breeding, resulting in a mosaic of genetic diversity. While the population size of 102 accessions used in this study is smaller than ideal for detecting numerous small‐effect loci typical of highly polygenic traits, the significant diversity within this panel (Figure 2) and the application of ML methods allowed for the identification of several genomic regions with potentially larger effects or complex interactions contributing to disease resistance and productivity. Future studies incorporating larger panels will be beneficial for increasing statistical power and detecting loci with smaller effects.

Our GWAS analyses, employing both BF and BT models, identified a suite of candidate genes associated with each of the four traits examined. This complexity highlights the potential utility of employing ML‐based GWAS, which may complement findings from traditional GWAS methods by potentially capturing complex effects (such as epistasis) or prioritizing loci differently, especially given the traits' polygenic nature. For instance, SNPs in proximity to a gene encoding a probable xyloglucan galactosyltransferase GT17 (*Tc02v2_g001090*, Figure 6, peak C, associated with all traits and flower cushion WBD) on chromosome 2 showed strong associations with flower cushion WBD resistance, PC1, and the trait product. Xyloglucans are major components of plant cell walls, and their modification can influence cell wall integrity and potentially impact pathogen penetration (Hématy et al., 2009). Another compelling novel candidate gene association emerging from our ML analysis involves galactinol‐sucrose galactosyltransferase/raffinose synthase (*Thecc1EG014511*, Figure 6, peak G, associated with pods with FPR), identified in the Matina dataset and associated with resistance to FPR, is known to accumulate during stress responses, implying a role in defense signaling (Chang et al., 2023). This aligns with the findings of Chang et al. (2023), who demonstrated that the raffinose synthase GhRFS6 in cotton interacts with GhOPR9, a 12‐oxo‐phytodienoic acid reductase involved in jasmonic acid (JA) biosynthesis, showing that GhRFS6 is upregulated during *Verticillium dahliae* infection and contributes to resistance by modulating JA signaling and cell wall properties.

We also identified a strong novel peak on chromosome 1 associated with a gene encoding ferrochelatase‐2 (*Tc01v2_g019770*, Figure 6, peak A, associated with all traits and flower cushion WBD), an enzyme involved in heme biosynthesis. Heme plays crucial roles in various cellular processes such as defense signaling, pointing to a potential role this gene may play in cacao's response to pathogens. This finding has parallels with recent work by Xu et al. (2024), who demonstrated that the RxLR effector Pi22922 from *Phytophthora infestans* targets potato StFC‐II, leading to increased susceptibility. While the specific effector may differ in our pathosystem, the involvement of FC‐II in modulating disease resistance appears to be conserved (Xu et al., 2024). Furthermore, the novel association of a gene encoding a putative exopolygalacturonase (*Tc01v2_g025400*, Figure 6, peak B, associated with pods with FPR) with multiple traits hints at the importance of cell wall degradation pathways in both productivity and disease resistance. This finding is particularly interesting in light of the work by Zhu et al. (2021) on *Verticillium dahliae*, a hemibiotrophic pathogen causing vascular wilt in a wide range of dicot hosts. They found that the deletion of a specific ExoPG gene in *V. dahliae* did not affect the pathogen's virulence on potato, despite the fact that this gene displayed increased expression in a highly aggressive isolate, proposing that the role of individual cell wall degrading enzymes (CWDEs) in pathogenicity can be complex and may involve compensatory mechanisms (Zhu et al., 2021). In our study, the association of an exopolygalacturonase with multiple traits hints at a broader role for this enzyme in cacao, possibly related to cell wall remodeling during growth and development, as well as in defense responses (Fry, 1995).

Additionally, the Matina‐based analysis using the trait product highlighted a novel candidate gene encoding an uncharacterized protein with a reverse transcriptase‐like domain (*Thecc1EG004701*, Figure 6, peak E, associated with all traits and flower cushion WBD). This association suggests a potential role for retroviral defense mechanisms or transposable element (TE) regulation in shaping overall plant performance, including disease resistance. As reviewed by Bennetzen and Wang (2014), TEs are key players in genomic novelty, influencing genome size, gene regulation, and creating new genes or pseudogenes. Additionally, a novel association near a leucine‐rich repeat (LRR)‐containing protein (*Thecc1EG006100*, Figure 6, peak F, associated with all traits and flower cushion WBD), often involved in pathogen recognition, was identified in the Matina dataset. This finding is consistent with the emphasis on the accurate identification of LRR proteins by Gottin et al. (2021) and further accentuates the potential importance of defense‐related pathways in influencing the composite traits. These results demonstrate how composite trait analyses can provide a more holistic perspective on the complex genetic architecture of cacao productivity, disease resistance, and overall well‐being; such interconnected pathways may be missed when analyzing individual traits separately.

The correlation analysis of SNP importance scores revealed varying degrees of consistency between the BF and BT models across different traits and genomes (Figure 7). Notably, the models exhibited strong concordance for the flower cushion WBD trait in both the Criollo (*r *= 0.91) and Matina (*r *= 0.82) genomes, a finding supported by the Venn diagram analysis (Figures 8d and 9d), which showed considerable overlap (16‐19 SNPs) in the top SNPs identified by both models for both cushion and branch WBD traits. For healthy pod rate, high correlation was observed in Criollo (*r* = 0.72), but it was moderate in Matina (*r *= 0.49). These findings, particularly the strong agreement both in correlation and top SNP overlap for WBD traits, bolster our confidence in the identified SNP‐trait associations for WBD, such as the robustly identified SNP chr5_29556644 found important for WBD by both models in Criollo (Figure 8d). However, the generally weaker correlations and limited overlap across different traits observed in the Venn diagrams (Figures 8a,b and 9a,b) suggest that distinct genetic architectures may underlie each trait, with limited evidence for pleiotropy within the scope of this study, although a few SNPs like chr5_29556644 (Criollo) and SCAFFOLD_3_22872047 (Matina) were highly ranked across multiple traits and models (Figures 8a and 9c).

Furthermore, the choice of reference genome influenced the results, as evidenced by the differences in SNP associations, candidate genes, and model correlations (e.g., healthy pod rate) identified when using the Criollo versus Matina genomes. These discrepancies likely stem from structural variations between the two genomes, differences in the SNP sets identified during mapping, or biases inherent in SNP discovery and the models themselves (Saxena et al., 2014). Future research employing a unified pangenome reference, such as the recently constructed *T. cacao* pangenome (Argout et al., 2023), which incorporates the genomic diversity of multiple cacao varieties, could help to resolve these inconsistencies and provide a more comprehensive understanding of the genetic basis of these important traits. Furthermore, benchmarking against known resistance loci revealed that several associations identified by our ML models occur on chromosomes previously implicated in resistance; for example, WBD associations were found on chromosomes 1, 4, 6, and 9, and FPR associations on chromosomes 1, 5, 6, 8, and 9 (Faleiro et al., 2006; Lanaud et al., 2009; McElroy et al., 2018; Motilal et al., 2016; Romero Navarro et al., 2017; Royaert et al., 2016), providing external support for our approach's ability to detect relevant genomic regions, although direct overlap with specific major QTLs reported in some studies was not observed.

Methodological evaluation using *K* = 5 cross‐validation revealed a common challenge in genomic studies with high dimensionality and limited sample size. While the BF and BT models demonstrated high explanatory power on training data (average R‐squared often > 0.70), their performance on validation sets was low and unstable (validation R‐squared typically < 0.20 and highly variable). This discrepancy highlights that while the models could fit the training data well, likely capturing complex relationships within this specific cohort, their ability to generalize and make accurate predictions on unseen data was limited by the sample size. This instability in validation underscores the rationale for utilizing the full dataset to obtain the most stable estimates of SNP importance for candidate gene discovery in this context, rather than relying on potentially misleading validation metrics. It is also important to acknowledge limitations inherent to using ML importance scores for association mapping. Unlike *p*‐values from linear models, the statistical properties of these importance scores, such as their distribution under the null hypothesis of no association, are less well characterized, making formal statistical threshold setting for controlling false positives challenging beyond pragmatic approaches like selecting the top N SNPs. Furthermore, because population structure was not explicitly modeled as a covariate, there is a potential risk that high importance scores could be partly driven by SNPs differentiating subpopulations that also happen to differ in mean phenotypic values, rather than solely reflecting causal association within the population. Furthermore, it is important to contextualize the specific candidate gene identifications. These genes were identified based on proximity to the SNPs with the highest importance scores. Given the SNP density in our study (average 39.8 kb) (Osorio‐Guarín et al., 2020), the identified SNP may be in linkage disequilibrium with the actual causal variant, which could reside within the identified gene or a nearby gene. Therefore, while the identified SNPs show strong association signals within this dataset, a direct comparison with results from traditional linear models (e.g., MLM, FarmCPU) on this specific dataset was beyond the scope of the current work but represents an important avenue for future research to fully delineate the advantages of each approach. Furthermore, both the predictive utility of these SNPs for MAS and the specific roles of the nearest candidate genes require experimental validation (e.g., expression analysis, functional studies), potentially through fine‐mapping in larger or independent populations. Addressing potential confounding due to population structure and developing robust statistical thresholds for ML‐based GWAS importance scores remain active areas of research.

In conclusion, this ML‐based GWAS has significantly expanded our understanding of the genetic architecture of productivity and disease resistance in cacao. We identified numerous candidate genes associated with these economically important traits, adding to the foundation laid by the original GWAS on this same collection by Osorio‐Guarín et al. (2020). Our analysis confirmed the potential importance of several genes identified in the previous study (Table 3) and brought to the forefront additional novel candidate genes, showcasing the potential of ML to reveal different facets of complex trait genetics. Practically, while ML‐GWAS approaches like the ones used here can potentially capture complex nonlinear or epistatic effects that may be missed by conventional linear models, they currently lack the standardized statistical validation frameworks (e.g., *p*‐value thresholds, FDR) and easily interpretable effect size estimates of the latter. Conventional GWAS remains crucial for identifying loci with strong additive effects, whereas ML‐GWAS serves as a valuable complementary tool for exploring complex genetic architectures and generating novel hypotheses. These findings provide a rich set of targets for future research within our program, including prioritizing candidate genes for functional validation through expression studies during pathogen infection and developing robust molecular markers from the most promising SNP associations for potential integration into MAS strategies in cacao breeding. Ultimately, combining diverse genomic approaches offers a powerful pathway to accelerate the development of improved, disease‐resistant cacao varieties.

## AUTHOR CONTRIBUTIONS

**Ezekiel Ahn**: Conceptualization; formal analysis; funding acquisition; investigation; methodology; project administration; software; supervision; validation; visualization; writing—original draft. **Sunchung Park**: Funding acquisition; investigation; methodology; validation; visualization; writing—review and editing. **Insuck Baek**: Funding acquisition; investigation; methodology; visualization; writing—review and editing. **Dongho Lee**: Validation; writing—review and editing. **Jishnu Bhatt**: Investigation; visualization; writing—review and editing. **Seunghyun Lim**: Investigation; methodology; writing—review and editing. **Jaehee Jang**: Investigation; methodology; writing—review and editing. **Dapeng Zhang**: Methodology; visualization; writing—review and editing. **Moon Kim**: Funding acquisition; methodology; resources; writing—review and editing. **Lyndel Meinhardt**: Funding acquisition; methodology; resources; writing—review and editing.

## CONFLICT OF INTEREST STATEMENT

The authors declare no conflicts of interest.

## Supporting information



Supplementary Material

Supplementary Material

## Data Availability

The phenotypic and genotypic datasets used in this study are available in the supplementary material of Osorio‐Guarín et al. (2020) in the journal *G3* (https://doi.org/10.1534/g3.120.401153). The results of our machine learning‐based genome‐wide association study are provided in the Supporting Information of this article. The file include tables with the SNP position in the context of both the Criollo and Matina reference genomes, and the SNP importance (portion) score for each trait and each machine learning model (Bootstrap Forest and BT). As our analysis employed machine learning algorithms, traditional *p*‐values were not generated. The SNP importance (portion) score serves as the measure of association strength.
