# Supplementary material for: Machine learning‐driven GWAS uncovers novel candidate genes for resistance to frosty pod rot and witches' broom disease in cacao
Source: Plant Genome. 2025 Jul 10;18(3):e70069. doi: 10.1002/tpg2.70069 (PMC12245736; doi:10.1002/tpg2.70069)
Supplement: Supplementary file 1 [file TPG2-18-e70069-s002.docx]

| **Trait** | **Chr/**  **Scaffold** | **SNP position** | **Nearest genes and function** | **GO annotation** | **Importance score (portion)** |
| --- | --- | --- | --- | --- | --- |
| **Bootstrap Forest** | | | | | |
| **All traits- Criollo** | 2 | 770436 | ***Tc02v2_g001090***  Probable xyloglucan galactosyltransferase GT17 (768191..770560) | - | 0.22 |
|  | 2 | 1064432 | ***Tc02v2_g001650***  Ion channel DMI1 (1060675..1066653) | - | 0.146 |
|  | 1 | 31472241 | *Tc01v2_g025400*  Putative exopolygalacturonase clone GBGE184 (31467814..31469699) | - | 0.0752 |
|  | 2 | 4523324 | ***Tc02v2_g007330***  MLO-like protein 4 (4521274..4527984) | - | 0.0688 |
|  | 1 | 21174545 | *Tc01v2_g019770*  Ferrochelatase-2, chloroplastic (21204419..21219868) | - | 0.0226 |
| **PC1- Criollo** | 5 | 29556644 | *Tc05v2_g015380*  Flavonol sulfotransferase-like (29555887..29564765) | - | 0.0654 |
|  | 2 | 1064432 | ***Tc02v2_g001650***  Ion channel DMI1 (1060675..1066653) | - | 0.0375 |
|  | 2 | 770436 | ***Tc02v2_g001090***  Probable xyloglucan galactosyltransferase GT17 (768191..770560) | - | 0.0375 |
|  | 1 | 31472241 | *Tc01v2_g025400*  Putative exopolygalacturonase clone GBGE184 (31467814..31469699) | - | 0.0347 |
|  | 3 | 29232746 | *Tc03v2_g015440*  NAD(P)-binding Rossmann-fold superfamily protein isoform 1 (29231573..29233074) | - | 0.031 |
| **All traits- Matina** | 2 | 1107779 | ***Thecc1EG006180***  Uncharacterized protein, castor and pollux, part of voltage-gated ion channel (1104002..1111419) | - | 0.1811 |
|  | 1 | 33051561 | *Thecc1EG004701*  Uncharacterized protein, Reverse transcriptase-like (RVT_3) // zinc-binding in reverse transcriptase (zf-RVT) (33049209..33052664) | - | 0.1383 |
|  | 2 | 794296 | *Thecc1EG006100*  Leucine-rich repeat-containing protein (794098..800964) | - | 0.1355 |
|  | 2 | 4567399 | ***Thecc1EG006939***  Seven transmembrane MLO family protein (4565183..4572400) | - | 0.0694 |
|  | 10 | 21386233 | *Thecc1EG044982*  Retrotransposon protein, RNA-directed DNA polymerase / Revertase // DNA-directed DNA polymerase / DNA-dependent DNA polymerase // Ribonuclease H / RNase H (21431241..21432083) | - | 0.0294 |
| **PC1- Matina** | 2 | 794296 | *Thecc1EG006100*  Leucine-rich repeat-containing protein (794098..800964) | - | 0.0449 |
|  | 2 | 1107779 | ***Thecc1EG006180***  Uncharacterized protein, castor and pollux, part of voltage-gated ion channel (1104002..1111419) | - | 0.0394 |
|  | 3 | 27055257 | *Thecc1EG015264*  NAD(P)-binding Rossmann-fold superfamily protein, 1.5.1.25 - Thiomorpholine-carboxylate dehydrogenase / Ketimine reductase (27053726..27055769) | - | 0.0362 |
|  | 8 | 2342743 | *Thecc1EG034244*  Clathrin adaptor complexes medium subunit family protein, AP-4 complex subunit mu-1 (AP4M1) (2077197..3268446) | - | 0.0234 |
|  | 5 | 21539335 | *Thecc1EG024023*  Uncharacterized protein, Chromo (CHRromatin Organisation MOdifier) domain containing (21306383..21649104) | - | 0.0208 |
| **Boosted Tree** | | | | | |
| **All traits- Criollo** | 1 | 31472241 | *Tc01v2_g025400*  Putative exopolygalacturonase clone GBGE184 (31467814..31469699) | - | 0.237 |
|  | 8 | 9354435 | *Tc08v2_g012500*  Hypothetical protein (9385538..9387618) | - | 0.1977 |
|  | 2 | 1064432 | ***Tc02v2_g001650***  Ion channel DMI1 (1060675..1066653) | - | 0.1664 |
|  | 2 | 770436 | ***Tc02v2_g001090***  Probable xyloglucan galactosyltransferase GT17 (768191..770560) | - | 0.1416 |
|  | 1 | 23747515 | *Tc01v2_g020200*  Uncharacterized protein (23778724..23781550) | - | 0.0744 |
| **PC1- Criollo** | 5 | 29556644 | *Tc05v2_g015380*  Flavonol sulfotransferase-like (29555887..29564765) | - | 0.1842 |
|  | 3 | 29232746 | *Tc03v2_g015440*  NAD(P)-binding Rossmann-fold superfamily protein isoform 1 (29231573..29233074) | - | 0.088 |
|  | 5 | 20862488, 20862417 | *Tc05v2_g010980*  Uncharacterized protein (21001814..21002302) | Nucleosomes wrap and compact DNA into chromatin | 0.0498 |
|  | 2 | 1064432 | ***Tc02v2_g001650***  Ion channel DMI1 (1060675..1066653) | - | 0.0465 |
|  | 2 | 770436 | ***Tc02v2_g001090***  Probable xyloglucan galactosyltransferase GT17 (768191..770560) | - | 0.0366 |
| **All traits- Matina** | 2 | 794296 | *Thecc1EG006100*  Leucine-rich repeat-containing protein (794098..800964) | - | 0.3089 |
|  | 2 | 1107779 | ***Thecc1EG006180***  Uncharacterized protein, castor and pollux, part of voltage-gated ion channel (1104002..1111419) | - | 0.2088 |
|  | 1 | 33051561 | *Thecc1EG004701*  Uncharacterized protein, reverse transcriptase-like (RVT_3) // zinc-binding in reverse transcriptase (zf-RVT) (33049209..33052664) | - | 0.1291 |
|  | 2 | 4567399 | ***Thecc1EG006939***  Seven transmembrane MLO family protein (4565183..4572400) | - | 0.0624 |
|  | 2 | 32406699 | *Thecc1EG010305*  Trichome birefringence-like 27, Protein altered xyloglucan 4 (32397560..32400435) | - | 0.0479 |
| **PC1- Matina** | 8 | 2342743 | *Thecc1EG034244*  Clathrin adaptor complexes medium subunit family protein, AP-4 complex subunit mu-1 (AP4M1) (2077197..3268446) | - | 0.1654 |
|  | 3 | 23882532 | *Thecc1EG014692*  Ribosomal protein L7Ae/L30e/S12e/Gadd45 family protein (23881262..23884077) | - | 0.1326 |
|  | 6 | 16208486 | *Thecc1EG028556*  Uncharacterized protein (16162795..16165220) | - | 0.0528 |
|  | 2 | 794296 | *Thecc1EG006100*  Leucine-rich repeat-containing protein (794098..800964) | - | 0.0499 |
|  | 2 | 1107779 | ***Thecc1EG006180***  Uncharacterized protein, castor and pollux, part of voltage-gated ion channel (1104002..1111419) | - | 0.0446 |

**Table S1. Top SNPs associated with composite measures of cacao productivity and disease resistance from Bootstrap Forest and Boosted Tree GWAS.** Composite measures are the product of four traits and their first principal component (PC1). Analyses were performed using the Criollo and Matina reference genomes. Table includes SNP identifier, chromosome/scaffold, position, nearest gene (with genomic location), putative gene function, and Gene Ontology (GO) annotation (if available). Genes in bold were also detected in the original GWAS conducted by Osorio-Guarín et al. [(2020)](https://www.zotero.org/google-docs/?10p4NC) on the same dataset.
